# Methods for conceptualising ‘visual ability’ as a measurable construct in children with cerebral palsy

**DOI:** 10.1186/s12874-017-0316-6

**Published:** 2017-03-21

**Authors:** Belinda Deramore Denver, Margareta Adolfsson, Elspeth Froude, Peter Rosenbaum, Christine Imms

**Affiliations:** 1 Australian Catholic University, School of Allied Health, Level 9, 33 Berry Street, North Sydney, NSW 2060 Australia; 2 Jönköping University, CHILD, School of Education and Communication, Box 1026, 551 11 Jönköping, Sweden; 3 McMaster University, IAHS Building, Room 408, 1400 Main Street West, Hamilton, ON L8S 1C7 Canada; 4 Australian Catholic University, School of Allied Health, Level 2, Daniel Mannix Building, 17 Young Street, Fitzroy, VIC 3065 Australia

## Abstract

**Background:**

Vision influences functioning and disability of children with cerebral palsy, so there is a growing need for psychometrically robust tools to advance assessment of children’s vision abilities in clinical practice and research. Vision is a complex construct, and in the absence of clarity about this construct it is challenging to know whether valid, reliable measures exist. This study reports a method for conceptualising ‘visual ability’ as a measurable construct.

**Methods:**

Using the items from 19 assessment tools previously identified in a systematic review, this study used a two-phase process: first, deductive content analysis linked items to the International Classification of Functioning, Disability and Health - Child and Youth version (ICF-CY), and second, vision-specific ‘Activity’-level items were explored using inductive thematic analysis.

**Results:**

The linking and content analysis identified that existing assessment tools are measuring vision across the ICF-CY domains of Body Functions, Activities and Participation, and Environmental and Personal Factors. Items specifically coded to vision at the Activity level were defined as measuring ‘how vision is used’, and these items form the basis of the conceptualisation that ‘visual ability’ is measurable as a single construct.

The thematic analysis led to the identification of 3 categories containing 13 themes that reflect a child’s observable visual behaviours. Seven abilities reflect how a child uses vision: responds or reacts, initiates, maintains or sustains looking, changes or shifts looking, searches, locates or finds, and follows. Four interactions reflect the contexts in which a child uses their vision to purposefully interact: watches and visually interacts with people and faces, objects, over distance, and with hands. Finally, two themes reflect a child’s overall use of vision in daily activities: frequency of use, and efficiency of use.

**Conclusions:**

This study demonstrates an approach to exploring and explaining a complex topic utilising World Health Organization language and building on existing research. Despite the complexity of vision, the concept of ‘how vision is used’ can be clearly defined as a measurable construct at the Activity level of the ICF-CY. This study has identified observable visual behaviours that may be developed into items assessing how vision is used in daily activities.

**Electronic supplementary material:**

The online version of this article (doi:10.1186/s12874-017-0316-6) contains supplementary material, which is available to authorized users.

## Background

Vision is an important construct to measure in children with cerebral palsy for both health care research and clinical practice. The primary motor disorder of cerebral palsy may be accompanied by additional impairments including vision [1], and there is growing evidence of the relationship between vision and various aspects of functioning [2–6]. This is not surprising as visual skills play an important role in development for all children, and the absence of, or limitations in, vision are known to impact development and functioning [7]. Children with cerebral palsy may be diagnosed with visual impairment at the ocular (eye) or cerebral/cortical (brain) level. One recent publication reported a prevalence of ‘some visual impairment’ in 36% of the population, and ‘functional blindness’ in 6% [6]. Information on the rates of visual impairments (ocular or cerebral) vary greatly in the literature [8]; however, it is likely that vision impacts outcomes for at least some children with cerebral palsy and their families.

Research in this area is expanding, but there are knowledge gaps and complexities to the assessment and management of vision for children with cerebral palsy [9–11]. Although valid and reliable assessment practices are required to evaluate and establish the effectiveness of interventions, there is currently limited clarity on *what* to measure, *how* to measure, *when* to measure and *who* should be measuring vision-related constructs for children with cerebral palsy. In the absence of clarity about the construct to be measured (i.e., the ‘what’), it is challenging to answer the question of whether a measure exists to answer clinical and research questions; this in turn can impact clinical and research outcomes [12]. A prerequisite to instrumentation and measurement is to determine what concept(s) is (are) to be measured, and how to translate the concept into measureable phenomena [13]. In this paper the phase of defining and understanding the construct to be measured is referred to as *conceptualisation*.

**Fig. 1:**
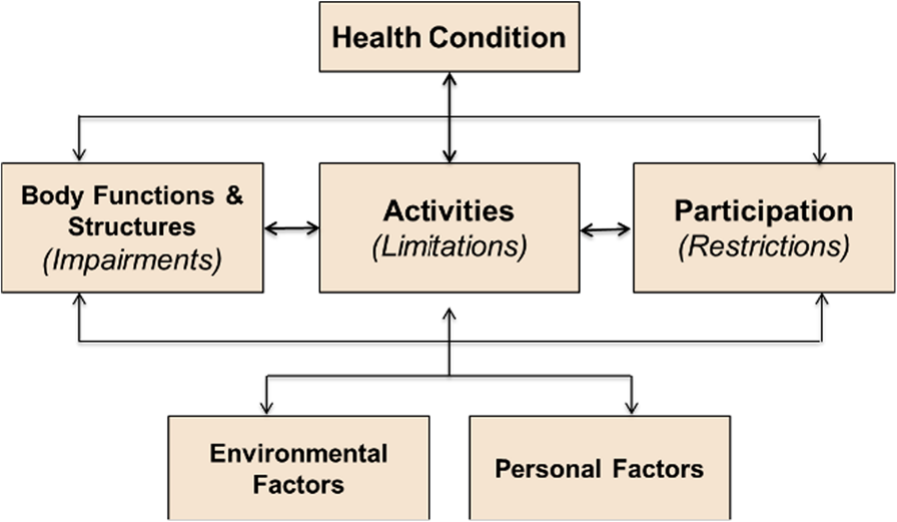
Framework of Functioning, Disability and Health (ICF and ICF-CY) [14, 15]

Vision is a complex construct, and its influence can be considered from multiple perspectives. These include how effectively a child’s eyes work, how well the child understands and interprets what they see, and how well vision is used in daily activities. The World Health Organization’s (WHO) International Classification of Functioning, Disability and Health (ICF) (2001) and the Child and Youth version (ICF-CY) (2007) provide a framework that can be used to consider functioning and disability, including vision, from a dynamic bio-psychosocial perspective [14, 15]. This framework (Fig. 1) includes four domains: (1) Body Functions and Structures; (2) Activities and Participation; (3) Personal Factors; and (4) Environmental Factors. The ability of a child to function is the outcome of a dynamic interaction among elements of these domains and contexts [15]. Within the ICF-CY, the concept of vision is represented at the Body Function level (*Seeing functions* and *Perceptual functions*) and the Activity level (*‘purposeful use of vision’*). These vision-related concepts interact in a process that is influenced by other factors including cognitive skills, motor abilities and aspects of the environment, and together they contribute to an individual’s overall level or functioning or disability.

Our recent systematic review on the measurement of visual ability in children with cerebral palsy focused on identifying tools assessing “vision that describes a child’s functioning at the Activity and Participation domain of the ICF-CY” p. 1018 [10]. This focus was driven by the need for clinicians to provide interventions at the Activity level, and the need for clinicians and researchers to have psychometrically robust methods to measure the effects of interventions. Measurement at the Activity level – that is, of ‘visual ability’ – is required to eliminate the need to make inferences or assumptions about levels of *functioning* in daily activities from an assessment limited to a Body Function (impairment) level e.g., visual acuity. Inclusion criteria for the systematic review were measures “addressing visual ability when the focus of the vision measurement was at the Activities and Participation domain of the ICF” p. 1019 [10], and the review included any tool designed or described as measuring “functional vision”. The systematic review did not identify an existing psychometrically valid and reliable tool that could be used. Findings also suggested that attributes included in existing assessment tools were conceptually varied and may not be limited to the assessment of how vision is used. From the review it was not possible to make a decision as to whether an existing tool could be modified by researchers [12], or whether a new assessment specific to how a child uses their vision in daily activities was required [10]. Thus, the need for an additional conceptual study was identified. The current study expands on the systematic review by analysing the content of identified tools at an item level. Content analysis was beyond the scope and inclusion criteria of the previous systematic review; however, it is critical that a measurement concept be clearly defined and understood before determining what, when and how to measure a phenomenon. The detailed content analysis in this study enables the important step whereby attributes can be identified and established as indicators of how visual ability can be measured [13]. This process supports the overall goal of this research program, namely to identify an approach to the assessment of visual ability or to generate items for the development of a new measure.

The systematic review defined *visual ability* as “how someone performs in vision-related activities” (p. 1019) [10]; the aim of the current study was to explore the ways that existing assessment tools conceptualised this as a construct at the Activity level of the ICF-CY. The specific research questions addressed were: (1) What ICF-CY constructs do items in identified assessment tools measure? (2) How can items that specifically assess vision at the Activity level of the ICF-CY be described in terms of what they measure? (3) What observable behaviours indicate levels of visual ability in assessment tools for children with cerebral palsy?

The study was conducted in two parts. Part I identified the *content* of measures in previously identified tools that assess vision at the Activity level of the ICF-CY. Part II identified and analysed the visual ability *themes* in that content. The goal was to identify assessments, or assessment items, to inform the future development of a valid visual ability assessment. This paper reports on the conceptualisation process used in this instrumentation research.

## Method

**Fig. 2:**
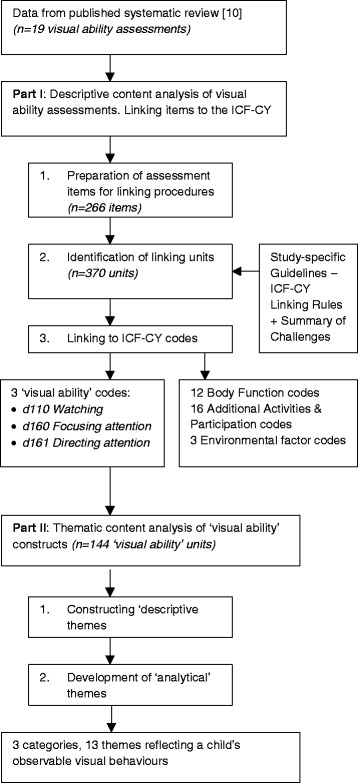
Flow diagram describing methodological process and results

This two-part qualitative study used both descriptive content and thematic content analysis, and the sequential process is illustrated in Fig. 2. Our earlier systematic review [10] utilised a rigorous process to identify 19 assessment tools containing 266 items that formed the units for analysis in this study. Details of the assessments tools, including purpose, format, psychometric properties and limitations, are described in detail in the systematic review [10]. The 19 assessments are variable in their purpose, including screening for CVI (e.g., [16]), developmental assessment (e.g., [17]), and assessment of daily visual performance (e.g., [18]). All assessments have been developed for, or used with children (0–18 years) with cerebral palsy or a diagnosis suggestive of cerebral palsy. All 19 assessments are included in this study as the focus was to capture the constructs measured by assessment tools, rather than how well visual ability was measured. The type of content and number of items, scales or questions are provided for all assessments in Table 1.

**Table 1:** Summary of included visual ability assessment tools

					ICF-CY Codes (N)^b^				Visual ability constructs^g^ N (%)
Assessment tool	Ref	Type of content^a^	Items/Scales	Linking units (N)	BF/BS^c^	ACT/PART^d^	ENV^e^	Other/Not coded^f^	
ABCDEFV	[35]	Clinical	22 Tests	28	20	8	0	0	6 (21.4)
Alimovic	[42]	Patient oriented	2 Scales	5	0	5	0	0	4 (80)
CAS	[33]	Clinical	33 Visual development items	41	14	26	0	1	18 (43.9)
CVI Q	[16]	Patient oriented	46 Items	56	11	41	0	6	28 (50)
CVI R	[31]	Clinical & Patient oriented	10 Characteristics	12	3	11	0	0	8 (66.7)
			1 Scale	1	0	1	0	0	1 (100)
EDVA	[32]	Clinical	7 Test Items	7	3	4	0	0	4 (57.1)
FVQ	[18]	Patient oriented	26 Questions	34	5	30	0	0	23 (67.6)
Hoyt	[43]	Patient oriented	1 Scale	2	1	1	0	0	1 (50)
HSCS-PS	[38]	Patient oriented	1 Vision Sub-scale	4	2	1	1	0	1 (25)
HUI-III	[44]	Patient oriented	1 Vision Sub-scale	5	2	2	1	0	1 (20)
IDP	[45]	Patient oriented	Visual competence 1 Scale	7	5	2	0	0	1 (14.3)
LVC	[30]	Clinical	8 Tests	10	2	8	0	0	4 (40)
PreViAs	[24]	Patient oriented	30 Questions	41	13	27	0	1	12 (29.3)
Short CVI Q	[34]	Patient oriented	12 Questions	15	5	10	0	0	4 (26.7)
SoGS	[17]	Clinical	22 Visual skill Items	25	13	12	0	0	10 (40)
VAP-CAP	[37]	Clinical	19 Items	34	22	12	0	0	10 (29.4)
VSI	[36]	Patient oriented	22 Items	35	11	17	3	3	6 (17.1)
Wong	[46]	Patient oriented	1 Scale	2	1	1	0	0	1 (50)
15-D	[47]	Patient oriented	1 Vision Sub-scale	6	1	3	2	0	1 (16.7)

*ABCDEFV* Atkinson Battery for Child Development for Examining Functional Vision, *CAS* Callier Azusa Scale, *CVI Q* CVI Questionnaire, *CVI R* CVI Range, *EDVA* Erhardt Developmental Visual Assessment, *FVQ* Functional Visual Questionnaire, *HSCS-PS* Health Status Classification System – Preschool, Vision scale, *HUI-III* Health Utilities Index – Mark III, Vision Scale, *IDP* Institutes’ Developmental Profile – Visual Competence Scale, *LVC* Low Vision Checklist, *PreViAs* Preverbal Visual Assessment, *Short CVI Q* Short CVI Questionnaire, *SoGS* Schedule of Growing Skills, Visual skills domain, *VAP-CAP* Visual Assessment Procedure – Capacity, Attention, and Processing, *VSI* Visual Skills Inventory, *15-D* 15-Dimension Questionnaire, Vision scale

^a^Type of assessment determines type of information to be linked: patient-oriented measure (self-report, caregiver report or health professional reported) or clinical assessment; ^b^Number of domain codes may equal more than the number linking units as some linking units were given two codes; ^c^Examples of constructs linked to Body Functions: seeing functions (visual acuity, visual field, and the ability to sense light, form, shape and colour, and eye functions), mental functions (orientation, memory, response time, visual perception and discrimination, visuospatial perception, knowledge and application of knowledge, recognition and object permanence), hearing functions, and neuromuscular functions such as reflexes and eye-hand coordination; ^d^Activities and Participation codes are expanded in Table 3; ^e^Environmental factors include supports or barriers of adapted products including large print or glasses/contacts, light in the environment, or people providing support; ^f^Other includes personal factors such as a child’s interest or mood, the use of compensatory strategies, and interventions such as patching; ^g^ Number of ‘visual ability’ constructs is total number of linking units coded to the visual ability codes (d110 Watching, d160 Focusing attention, d161 Directing attention) as % of the total linking units

### Part I: process of linking visual ability assessments to the ICF-CY

Part I provided a descriptive content analysis of previously identified visual ability assessment items utilising established methodology for the linking of measurement tools to the ICF-CY. The ICF-CY classification contains categories and codes in two sections. The first part refers to functioning and disability and includes Body Functions (b) and Body Structures (s), and Activities and Participation (d). The second part refers to Contextual Factors and includes Environmental Factors (e) and Personal Factors [15]. The classification is an alphanumeric system. The letters b, s, d, and e refer to the category or domain of the classification and are followed by a numeric code that starts with the chapter number (a single digit), followed by the second level (two digits), and the third and fourth levels (one digit each) [15]. An example from the Activities and Participation domain is as follows:

- d1 Learning and applying knowledge (first or chapter level)
- d160 Focusing attention (second level)
- d1600 Focusing attention on the human touch, face and voice (third level)

Published *ICF Linking Rules* detail the steps for the process of linking measurement tools to the classification system. These rules include two key stages: 1) identification of ‘linking units’, and 2) linking the units to ICF-CY codes [19–21]. Table 2 summarises published rules, together with examples specific to this study, and was used by the authors to undertake the process. Linking methodology has previously been used to compare and contrast information from outcome measures for the purpose of clarity about constructs (e.g., upper limb measures for children with cerebral palsy [22]).

**Table 2:** Study specific ICF-CY linking rules

Identification of linking units	
i.	Determine the type of information to be linked: *patient-oriented measure* (self-report, caregiver report, or health professional reported) or *clinical assessment*.
ii.	Identify linking unit(s). The linking unit of a measure answers the question: What is the item about? The names of measures, the instructions, and subscale titles provide useful information to define the linking units. e.g., *Item 17 from the CVI Questionnaire asks whether the child “Sits right in front of the television”. This item needs to be considered in the context of being an item in a measure screening for cerebral visual impairment. The item falls in the section of ‘Visual attitude’ and the subscale of ‘visual attention’. This item is not about ‘sitting’.* For Patient-oriented measures: • Refer to the item as it appears in the questionnaire • Identify response options of items that contain linking unit(s) For Clinical assessments: • Refer to the aim of the clinical assessment • Consider that the linking unit may change depending on the context in which the clinical assessment is used.
iii.	Identify any relationship between concepts: when there are more than two linking units the relationship between the units is also provided. e.g., *Item 21 in the Functional Visual Questionnaire asks whether the child “Looks at a toy or object while reaching/moving hand towards it”. This item is about looking ‘whilst’ reaching. The relationship should be recorded.*
Linking of linking units to the ICF-CY	
a.	Select the appropriate code(s) to describe the linking unit: Is the linking unit an element of Body Functions, Body Structures, Activities and Participation, or Environmental factors? Which chapter within the selected domain is the most appropriate? Which category within the selected chapter is the most precise?
b.	If the content of an item is not explicitly named in the corresponding ICF-CY category, then the “other specified” is linked. This code allows for coding of functioning that is not included within any of the other specific categories. When an “other specified” code is used, the specification has to be annotated.
c.	If the content of an item is insufficient to permit assignment of a more specific category, the “unspecified” is linked. The code has the same meaning as the second- or third-level term immediately above (b), without any additional information. i.e., Use d199 Learning and applying knowledge, unspecified rather than d1 Learning and applying knowledge
d.	If the linking unit is an element of ‘Health condition’ the code HC is used.
e.	If the linking unit is an element of ‘Personal factors’ it would be considered to have a positive or negative influence on disability and functioning. To determine if a linking unit is a Personal factor ask: Can the linking unit be impaired, restricted or limited? If no, it is a personal factor.
f.	If the content of an item is unclear or too general to permit assignment of any category or component, the “nondefinable” (nd) is used. The perspective is documented as General Health (nd-gh), Quality of life (nd-qol), Physical health (nd-ph), Mental health (nd-mh), or Life satisfaction (nd-s).
g.	If the linking unit is not a Health condition, Body function/body structure, Activity, Participation, Environmental factor or Personal factor, it is “Not covered” (nc).

The deductive linking process was completed by researchers with good knowledge of the concepts, definitions and structure of the ICF-CY. The first author (BD) is an occupational therapist with experience working with children with cerebral palsy and vision impairment, and had acquired relevant knowledge using the eLearning tool developed by the World Health Organization [23]. The second and last authors (MA and CI) are both knowledgeable in the ICF-CY and linking methodology, and all authors have clinical experience with the cerebral palsy population.

The first author initially prepared the data for linking by entering all 266 items from the 19 measures into a linking extraction table. Next, items were analysed independently by two authors (BD and either MA or CI) to identify linking units (‘what the item is about’). Items were analysed for both main and additional concepts; this was done at an item and response level for patient-oriented measures, and by considering the aim in clinical assessments. This process was complex, with most measures containing some items whose meaning was unclear, making it difficult to know what the item was about, and as a result, the identification of linking units and ICF-CY codes was inconsistent between linkers*.* For example, Item 3 from the Preverbal Visual Assessment (PreViAs) asks “Is he/she able to look towards a sound source?” [24]. Different authors (linkers) considered that this item may be about ‘looking’, ‘turning to look’, ‘hearing a sound’ or ‘sound localisation’. Five iterative rounds of independent linking were subsequently undertaken using a process of constant review, comparison and discussion until consensus was reached.

Consistent consensus-based decisions were made possible when a set of study-specific guidelines was developed from notes on discussions and refined continuously as suggested by other authors [25–28]. The guidelines are a summary of ICF Linking Rules [19, 20, 23] annotated with study-specific examples, in addition to a summary of solutions to commonly occurring challenges specific to this study (available in Additional file 1). Throughout the linking process, the guidelines were used to improve the consistency of the approach. Once consensus-based decisions could be reached by the first and second author using the guidelines, the first author completed the linking for all assessments. Units were linked to the most precise code in the ICF-CY, however most results are reported and discussed at the second level.

To present the results a tabulated descriptive summary is provided for assessment tools including details of the assessment tool and type of information to be linked, number of items and linking units, and the number of linking units for each of the ICF-CY domains. The number of linking units determined to be measuring ‘visual ability’ is presented for each assessment tool, and details of all Activities- and Participation-level codes at a two-level classification are presented to illustrate what constructs are measured by existing assessment tools. Details of the Body Function and Environmental factor codes are available in Additional file 2.

### Part II: process of establishing ‘visual ability’ themes

Part II included thematic content analysis undertaken in two steps [29] to examine the 128 items that linked to specific codes identified in Part I as vision in the Activities and Participation domain. In addition to the ICF-CY code of *d110 Watching*, two additional second-level codes were commonly considered to be about the use of vision: *d160 Focusing attention*, and *d161 Directing attention*; however, care was taken in the analysis of items linked to these codes as they might not be exclusive to vision. The analysis involved (a) constructing ‘descriptive’ themes (e.g., ‘tracking’), followed by (b) the development of ‘analytical’ themes (e.g., ‘follows’). The results of this process were recorded in the same data management and extraction table used in Part I.

To construct descriptive themes, the first author (BD) immersed herself in the data and sought evidence for (1) verbs describing visual abilities, and (2) indicators, characteristics or specifications of different levels of visual ability. This decision was guided by the overarching aim of the study, namely that the results should inform the development of a new visual ability assessment. It was determined that words describing how vision is used (e.g., verbs) would be essential to the development of an ability measure. Table 3 provides four examples of the inductive process of constructing descriptive themes.

**Table 3:** Example of process to identify linking units and ICF-CY codes (Part I) and ‘visual ability’ themes & categories (Part II)

Part I				Part II			
				Descriptive theme		Analytical theme	
Measure	Item	Linking unit^a^	ICF-CY code^b^	Descriptive word for visual ability	Indicator of visual ability	Theme: Observable visual behaviour	Category of visual ability behaviour
CVI Q [16]	Manipulates an object rather than look at it *(Item 40, Other senses domain)*	Use of other senses	d110 Watching d1201 Touching^c^	Look	Look at object Manipulate rather than look (other senses)	Watches and/or visually interacts with objects Frequency of use of vision in activities	Interactions Use of vision
FVQ [18]	Tracks an object/toy *(Item 2)*	Tracking	d110 Watching	Tracking	Tracks an object/toy	Follows	Abilities
						Watches and/or visually interacts with objects	Interactions
PreViAs [24]	Is he/she able to look towards a sound source? *(Item 3)*	Looking toward a sound source^d^	d110 Watching b2302 Localisation of a sound source	Look	Looks toward sound source	Searches	Abilities
VSI [36]	Does your child reach for a large, bright, silent object? *(Item 17)*	Reaching	d4452 Reaching	n/a			

^a^Linking unit = What is the item about?; ^b^Only assessment items which have been linked to an ICF-CY ‘visual ability’ code of d*110 Watching*, *d160 Focusing attention* or *d161 Directing attention* are included in Part II; ^c^This is an example where the exact term in the ICF-CY does not match the construct as described in the measure i.e., linked to *d1201 Touching* and not *d4402 Manipulating*; ^d^Example of an item where it was not easy to identify what the item was about e.g., is it about ‘Turning to look’?, ‘Hearing a sound’ or ‘Looking’

Analytical themes were developed by the first author (BD) from the descriptive themes by grouping similar verbs and indicators into clusters that could be identified using an over-arching label that reflected the ‘observable visual behaviour/s’. This stage was influenced by knowledge of the literature, research, and clinical practice in the area. The results were confirmed by the co-authors (EF and CI) independently analysing 15% of the items and discussing themes until consensus was reached. Short descriptions of theme clusters were written and validated by referring back to the items. A final step involved the grouping of similar themes into overarching categories that reflected all themes within the group. The process of developing analytical themes and combining these into categories is also illustrated in Table 3.

The results of Part II are reported using a narrative description of the analytical themes as visual behaviours observable in daily activity performance of children with cerebral palsy. The themes are presented under their categorical headings, along with examples or extracts from items, responses or instructions from visual ability assessment tools that contributed to their development. Examples from a range of assessment tools are utilised to assist with the transparency and trustworthiness of the findings and interpretations. In line with the overarching goal of establishing a method for assessing the visual ability of children with cerebral palsy, examples that represented the themes were selected from included tools to describe *ability*, rather than what a child *cannot do* (e.g., “…keep looking” rather than “cannot keep looking” CVI Q) [16].

Decision points throughout both phases of this research were regularly discussed among the authors, ensuring a peer review process aiming to increase the confirmability of the results.

## Results

### Part I: constructs measured by vision assessments

In total, 266 assessment items, scales or tests were included in the analysis of constructs measured by existing assessment tools, and 370 units were linked to the ICF-CY. Items were linked to constructs across the ICF-CY domains including Body Functions, Activities and Participation, Environmental factors and Personal factors (see Table 1). This study found that all 19 previously-identified assessments contained items and linking units that were linked to one of the specific codes identified as ‘visual ability’ codes (*d110 Watching*, *d160 Focusing attention*, and *d161 Directing attention*) (see Table 4), but in addition to measuring vision, an additional 16 second-level codes from the Activity and Participation domain were also identified as constructs within the assessment tools (e.g., d445 Hand and arm use, for items about reaching). These findings support the previous decision for inclusion of all 19 assessments in the systematic review, and also confirm that these tools include measurement of a variety of constructs. Whilst vision measurement is varied, occurring across the ICF-CY domains, the results suggest that vision measured using specific ‘visual ability’ items could result in measurement of a single construct, and further analysis was indicated.

**Table 4:** Activity and Participation ICF-CY categories identified in assessment tools

	Assessment tools with visual ability items^a^																			
ICF-CY Activities and Participation Chapters and Two-level classification^b^	ABCDEFV	Alimovic	CAS	CVI Q	CVI R	EDVA	FVQ	Hoyt	HSCS-PS	HUI-III	IDP	LVC	PreViAs	ShCVI Q	SoGS	VAP-CAP	VSI	Wong	15-D	N
d1 LEARNING AND APPLYING KNOWLEDGE																				
d110 Watching and/or d160 Focusing attention^c, d^	**X**	**X**	**X**	**X**	**X**	**X**	**X**	**X**	**X**	**X**	**X**	**X**	**X**	**X**	**X**	**X**	**X**	**X**	**X**	**19**
d120 Other purposeful sensing^e^				X	X		X										X			4
d130 Copying^f^			X										X							2
d131 Learning through actions with objects^g^	X		X										X							3
d161 Directing attention^h^	**X**	**X**		**X**																**3**
d166 Reading^i^										X	X			X					X	4
d170 Writing^j^													X							1
d2 GENERAL TASKS AND DEMANDS																				
d3 COMMUNICATION																				
d315 Communicating with – receiving – non-verbal messages^k^				X			X						X				X			4
d335 Producing nonverbal messages^l^							X						X		X					3
d350 Conversation^m^		X																		1
d4 MOBILITY																				
d440 Fine hand use^n^	X		X	X	X		X					X	X			X				8
d445 Hand and arm use^o^			X		X		X						X		X	X	X			7
d450 Walking^p^				X								X		X						3
d460 Moving around in different locations^q^												X							X	2
d499 Mobility, unspecified^r^			X	X																2
d5 SELF-CARE																				
d6 DOMESTIC LIFE																				
d7 INTERPERSONAL INTERACTIONS AND RELATIONSHIPS																				
d710 Basic interpersonal interactions^s^			X	X			X						X	X			X			6
d8 MAJOR LIFE AREAS																				
d880 Engagement in play^t^				X									X							2
d9 COMMUNITY, SOCIAL AND CIVIC LIFE																				
d920 Recreation and Leisure^u^				X																1

^a^Assessment tools identified in systematic review [10]; ^b^Only two-level classification codes linked to items from visual ability assessment tools are presented in this table; ^c^Items linked to codes *d110 Watching* and *d160 Focusing attention* are combined in this presentation due to difficulties in discriminating between the constructs, and the three concepts which represent the concept of ‘visual ability’ are presented in bold font; Examples of constructs linked to Activities and Participation codes: ^d^Focusing on or tracking a toy, ^e^Mouthing, touching and smelling, ^f^Imitation of facial expression, ^g^Relating two or more objects such as block building or posting, ^h^Keep looking, ^i^Reading crowded text, ^j^Scribble with pen on paper, ^k^Responds to/understands facial expressions, ^l^Smiles or demonstrates visual preference, ^m^Starting/sustaining visual communication, ^n^Picking up, Grasping or Manipulating object, ^o^Reaching for seen object, ^p^Walking around and over different surfaces and avoiding obstacles, ^q^Moving about +/− guidance, ^r^Moves to object, ^s^Appropriate use of eye contact and differentiation of familiar people/strangers, ^t^Play with objects, and ^u^Memory game

### Part II: analysis of ‘visual ability’ items

Thirteen analytical themes emerged from the data to describe items that specifically assess vision at the Activity level of the ICF-CY. These 13 themes are clustered into three categories that reflect a child’s observable visual behaviours (Table 5). The category *Abilities* includes seven themes reflecting how a child uses vision; *Interactions* includes four themes reflecting the contexts in which the child uses their vision to interact purposefully; and *Use of vision* includes two themes reflecting a child’s overall use of vision in daily activities. These results provide the conceptualisation of the construct ‘visual ability’.

**Table 5:** Categories and related themes reflecting how visual behaviours are described in assessment tools

I. Abilities	II. Interactions	III. Use of vision
1. Responds or reacts 2. Initiates 3. Maintains or sustains looking 4. Changes or shifts looking 5. Searches 6. Locates or finds 7. Follows	8. Watches and interacts visually with people/faces 9. Watches and interacts visually with objects 10. Watches and interacts visually over distances 11. Watches and interacts visually with hands	12. Frequency of use of vision in activities 13. Efficiency of use of vision in activities

### Category I: abilities

#### Responds/reacts

The first theme incorporates the basic visual ability of responding or reacting to visual stimuli, and utilises *observations of behaviours that suggest a child is responding, at some level, to visual information*. The theme is derived from items describing a wide range of responses or reactions and includes both purposeful and non-purposeful use of vision, and both passive and active responses.

…the light perception test is deemed positive if the patient shows some reaction to light, even high-intensity light…by moving his or her head, winking, or making a defensive or stopping movement (extract from LVC, Test 1 guidelines) [30].

Items that contributed to the development of this theme often appeared first in a measurement tool, and it is proposed that responding or reacting is a pre-requisite for other visual abilities i.e., if a child does not respond they will not be able to demonstrate other visual behaviours such as watching, finding, or following. Some items themed to ‘responds or reacts’ were additionally linked to b210 *Seeing functions* in Part I.

#### Initiates

This theme is about how quickly vision is used; the observable behaviour is *time to respond to visual information in a purposeful way*. Items contributing to this theme include descriptions of prompt or delayed responses.

Exhibits a delayed response to visual stimuli (FVQ, Question 6) [18].

#### Maintains/sustains looking

This theme is about how much or for how long a child keeps looking. The observable behaviour is *the purposeful use of vision for a length of time appropriate to the activity*.

…keep looking at objects or persons (extract from CVI Q, Item 9) [16].

Contextual information about type of visual stimuli or the environment where the visual behaviours occur reflects some of the variability in items about a child’s ability to maintain/sustain looking, and these facilitators or barriers also apply to the previous theme of ‘initiates’.

… brief fixations on movement and reflective materials; Movement continues to be an important factor to initiate visual attention; Movement not required for attention at near…(extract from CVI Characteristic - Need for movement, CVI R) [31].

#### Changes/shifts looking

This theme addresses whether the child can initiate a purposeful change or shift in looking between objects, people and/or the surrounding environment. The observable behaviour is *the child easily disengaging attention from one stimulus to look at another*.

…able to move the eyes quickly between two persons or two objects (extract from Question 4, PreViAs) [24].

Shifts gaze between targets in near and middle space accurately (extract from 5-month Pattern Component, Gaze Shift - Visual Release, EDVA) [32].

Items contributing to the theme suggest variations in the ability to shift gaze, and may include use of internal strategies (e.g., blinking to facilitate visual release) and/or the need for physical support to prompt or redirect looking behaviours.

#### Searches

This theme considers whether the child uses a process of visually searching, scanning and exploring in a purposeful way. Searching may or may not result in ‘finding’ the desired target – that is themed separately. The observable behaviour is *the self-initiated ability of the child to explore visually by moving their visual attention around the information in the visual environment for a goal-directed purpose*.

Visually seeks missing object or person (Item 9b, CAS) [33].

Looks around when entering a room (Question 25, FVQ) [18].

By definition, this theme is suggestive of prerequisite skills including initiation, the ability to interact with different stimuli including over distances, sustained looking or attention, and shifting between stimuli.

#### Locates/finds

The theme ‘locates/finds’ is about whether and how easily a child uses their vision to locate or find specific information. The observable behaviour is *successfully locating the specified or required visual information*.

Looks in correct place for fallen toy (Item 78, SoGS) [17].

Items that contribute to the development of this theme suggest that the ease with which a child locates or finds specific visual information may be impacted by the environmental context in which the behaviour occurs, including distance, background clutter, colour, low contrast/similar background, in addition to the prerequisite skills described under the ‘searches’ theme. Success in locating or finding a target are more likely to be observed if a child has good searching abilities.

…find his teddy bear (or equal) amongst other cuddly animals (extract from Item 33, CVI Q) [16].

…Finding parents or friends in a crowd (extract from Question 3, Short CVI Q) [34].

This theme was predominantly derived from assessment items designed to diagnose or screen for cerebral or cortical visual impairment (CVI), suggesting that locates/finds may contain significantly more cognitive requirements than some other abilities. In addition to items about locating or finding a person or object, this theme also included items about navigation.

…find his/her way to the classroom, in his house [familiar environments] (extract from Item 26, CVI Q) [16].

#### Follows

This theme, and the observable behaviour, *concerns whether and how effectively the child follows or tracks moving targets*. It was derived from items also contributing to other themes, including the types of stimuli that are followed, the distances at which following occurs, and how often a child demonstrates following behaviours. The abilities that are unique to this theme are the direction and extent (e.g., how far) of following behaviours, and the quality of the following with eyes and/or head.

…Either saccadic (jerky) tracking or smooth pursuit can be accepted but it should be noted which type of eye movement the child makes … For infants over 3 months, tracking should be easily elicited on the first trial in either direction, provided the child is reasonably attentive at the start of each trial (extract from procedure, Item 3, ABCDEFV) [35].

The content of items contributing to this theme, and the relationship between items in different themes, suggests that following has a number of prerequisite abilities including ‘sustains looking’. There is also a suggestion that ‘shifts looking’, ‘searches’ and ‘finds’ may result in successful performance (‘use of vision’) in the absence of the ability to follow.

### Category II: interactions

#### Watches and interacts visually with people & faces

The first ‘interaction’ theme describes whether the child watches or looks at people and faces; the observable behaviour is *purposeful looking at people and faces within everyday social interactions*.

…Generally no regard of the human face…Regards familiar faces when voice does not compete… Smiles at/regards familiar and new faces… Typical visual/social responses (extract from CVI Characteristic – Visual Complexity, CVI R) [31].

Focuses on a face when seated opposite him/her (Question 13, FVQ) [18].

The importance and relationship of this theme to a child’s overall functioning is evident when revisiting the items and codes analysed in Part I of this study where additional related concepts included the variables such as responding to facial expressions and recognising faces.

#### Watches and interacts visually with objects

This theme explores whether the child looks at objects (e.g., inanimate stimuli such as toys and books) and includes the range of objects with which the child watches or visually interacts. The observable behaviour is *the child’s purposeful response to the visual properties of objects*, in a manner which is appropriate to the child’s motor capacity and developmental level.

…reach for a drink bottle when you hold it in front of him/her…become excited but does not reach for the drink bottle (extract from Item 11, VSI) [36].

Looks at/focuses on pictures in a book or on a communication board (Item 19, FVQ) [18].

Limitations in the range of stimuli with which a child interacts visually are suggested by items describing the need for specific characteristics to facilitate looking e.g., sound, light, colour.

Requires an additional sensory modality (e.g. sound, touch, etc.) to focus on or respond to an object/toy (Question 7, FVQ) [18].

…Objects viewed are generally a single colour…(extract from CVI Characteristic – Color Preference, CVI R) [31].

#### Watches and interacts visually over distances

This theme is about whether the child watches/looks at visual information over a range of distances. The observable behaviours are *responses indicating that visual information has been experienced*. It is about seeing/using vision to experience information beyond the child’s immediate space, and the distance is considered in relation to the child’s age.

Visually attends in near space only … Visual attention extends beyond near space, up to 4 to 6 feet (extract from CVI Characteristic: Difficulty with distance viewing. CVI R) [31].

Watches movements of people at distances or out of window with interest (Item 79, SoGS) [17].

#### Watches and visually interacts – with hands

The next theme is about whether there is an interaction between the child and the manual actions of his/her hands, or the manual actions done by the hands of another person. The observable behaviour is *whether there is purposeful and effective use of this interaction in everyday activities*. Whilst it is acknowledged that children with cerebral palsy have varying manual abilities, the interaction between vision and manual actions is a strong theme.

…observe his/her own hands (extract from Question 6, PreViAs) [24].

Visually explores the toy whilst you turn it over: The child looks interested in the toy but either because of physical disability or tactile defensiveness can’t or won’t take the toy, but visually examines the toy as the adult turns it over (extract from response option, Item 5, Low Vision Assessment, VAP-CAP) [37].

Looks at a toy or object while reaching/moving hand towards it (Item 21, FVQ) [18].

The identification of relationships between linking units, as recommended in the ICF eLearning Tool [23], contributed significantly to this theme with many of the items contributing to this theme also being linked to another ICF-CY code (e.g., *d1201 Touching* or *d440 Fine hand use*).

### Category III: use of vision

#### Uses vision in activities – Frequency of use

This theme is about observations of *the overall frequency or ‘how often’ the child uses their visual abilities*. This theme is derived from items describing the consistency and reliability with which visual abilities are used in daily activities.

…Student functions with more consistent visual response…(extract from scoring, Rating I, Across CVI Characteristics, CVI R) [31].

Attention is fluctuating from moment to moment and from day to day (Item 10, CVI Q) [16].

This theme was also developed from items suggesting a low frequency of use of vision by referring to the use of senses other than vision (e.g., listening, mouthing, touching, smelling, or tasting) when vision could be used.

Manipulates an object rather than look at it (Item 40, CVI Q) [16].

#### Uses vision in activities – Efficiency of use

The final theme is about the efficiency with which vision is used in daily functioning. The observable behaviours are *how independently and easily a child has success when performing in vision-related activities*. Items contributing to this theme describe how performance in vision-related activities is affected by limited visual functions, and describe limitations in performance related to the need for assistance, guidance, time or prompting, a reduced level of independence, or difficulty in performance. As such, items contributing to this theme were commonly linked to codes in addition to the visual ability code in Part I, such as *b1561 Visual perception, b210 Seeing functions*, and *e1251 Assistive products and technology for communication*.

…able to see well enough to recognise small objects and familiar people at a distance…Sees objects close to oneself - e.g. at arm’s length, but has visual limitations at distance, even with glasses (extract from Vision (ability to see) subscale, HSCS-PS) [38].

## Discussion

This paper presents a methodological approach applicable to research conundrums where definition and understanding of a complex issue are required. Our example involves the initial stages in instrumentation research to establish ‘vision use’ as a construct that is measurable in children with motor impairments. By utilising a two-part process this study demonstrates an approach to conceptualise complex constructs and operationalise how a concept will be measured. In this study the WHO’s ICF-CY provided a framework for conceptualising a complex construct utilising terminology that has been endorsed world-wide [15], increasing the transferability of both the methods and findings. The outcome from this work is a conceptualisation of visual ability that is grounded in a common language and builds on, and takes advantage of, the work of previous researchers. It is an approach that other healthcare researchers, clinicians and policy makers are encouraged to consider when clarity is sought regarding complex or unclear constructs.

In the first phase of this study a deductive and explanatory method established ‘visual ability’ within the conceptual framework of the ICF-CY. The process built upon the focus of vision measured at the Activities and Participation level of the ICF-CY previously presented in a systematic review [10], and developed a refined definition of ‘visual ability’ as a construct measureable *within* the Activity level of the ICF-CY as ‘how vision is used’. This finding arose from linking procedures that identified that existing assessment tools measuring visual ability in fact measure a wide range of constructs. This demonstrates the complexity and multidimensionality of ‘vision’, and provides valuable information about the need to define clearly which component(s) of functioning is (are) being measured at any given time. At an item level, existing visual ability assessment tools are measuring constructs across the ICF-CY framework, and these findings support the need for the development of a discrete assessment tool that measures ‘visual ability’.

Whilst the ICF-CY provides a strong framework from which to develop the conceptualisation of visual ability, the process of linking items to the classification in this study was not straightforward. It is proposed that issues identified during linking in this study regarding ‘what an item is about’ likely reflect problems utilising the existing measurement tools in clinical practice and research. If the authors of this paper could not reliably link items, it is reasonable to assume that parents and clinicians may also be unlikely to respond consistently to items, thus potentially impacting both the reliability and validity of measurement. The development of study-specific guidelines was an important step in this study to establish trustworthiness in the findings, and a summary of key challenges encountered during the linking process is provided in Additional file 1. This information will be useful to researchers wishing to apply these methods in the future.

It must be recognised that the study results may not reflect the original intent of the authors of included measures. Linking the content of existing tools to the ICF-CY was completed in this study as one step in the methodological process of defining the concept of ‘visual ability’ and its place within the larger conceptual framework. The process of making conceptual distinctions within measurement tools and how this is important for content validity has previously been reported in quality of life research [39].

In the second phase, the application of an inductive and exploratory method resulted in a description of visual ability using 13 behaviours observable during typical daily activities. These behaviours are not new, but it is proposed that the act of identifying and describing these themes forms the step of item generation for a new assessment tool as this research moves from conceptualisation of visual ability to a measurement development phase. The analytical process and interpretation in this study also suggest the possibility of a hierarchy of visual abilities within the identified behaviours, that is, that careful ordering of the behaviours may reveal how a child functions in vision-related activities. This is a finding which could be explored in future instrumentation work using Item Response Theory [40].

Whilst the results of this study provide key foundational information for the development of an assessment of visual abilities in children with cerebral palsy, they are not yet operationalised in a measure. The observable behaviours are expected to be of interest to a wide range of researchers and clinicians, however they require further revision, development and validation before they can be considered an ‘assessment’. In their current format the results of this study may only provide guidance to practitioners in relation to their informal observations of visual abilities in children, and will likely inform discussion and future research. The previously published systematic review provides a summary of currently available assessment options and recommendations for assessing children with cerebral palsy. However, it is important to note that the assessment tools reviewed in the systematic review do not measure the construct of visual ability as conceptualised in this methods paper.

Because this study used existing measures as the unit of analyses, whether all themes identified within this study are relevant, and whether they represent a comprehensive set of items about vision use, is an empirical question that requires further research. It is imperative that individuals with cerebral palsy, parents and carers, and the professionals who work clinically with the population contribute to future development of the visual ability construct, and the way it is measured [41]. It will be important to confirm the relevance of the observable behaviours across the diverse cerebral palsy population including people of different age groups, gross motor, manual and cognitive abilities. It is also likely that the definition of visual ability established in this study could be applicable to a range of health conditions other than cerebral palsy, however further investigation of the validity of this premise would be required.

## Conclusion

Despite the complexity of vision, the concept of ‘how vision is used’ can be clearly defined as a measurable construct within the Activity level of the ICF-CY, so discrete measurement of this construct appears feasible. This construct is labelled ‘visual ability’, and this study has identified observable visual behaviours that may be developed into items assessing how vision is used in daily activities. The approach used in this study to explain and explore a complex construct may be useful in other health care research. Future research is required to confirm the results of this study and expand the findings through further instrumentation research. It is now planned that a tool be developed and validated to assess the construct of visual ability in children with cerebral palsy, and then used to establish effective interventions to optimise how vision is used.

## Additional files

Additional file 1: Study-Specific Guidelines: ICF-CY Linking Rules and Challenges. This file contains a summary of the ICF Linking Rules annotated with study-specific examples and a summary of solutions to commonly occurring challenges from Part I – Linking visual ability assessments to the ICF-CY. (DOCX 20 kb)
Additional file 2: Results table for Body Function and Environmental factor codes. This file contains the tabulated results for assessment items linked to Body Function and Environmental factor codes. These results are not pertinent to Phase II in this study. (DOCX 21 kb)
