# Supplementary material for: Methods for conceptualising ‘visual ability’ as a measurable construct in children with cerebral palsy
Source: BMC Med Res Methodol. 2017 Mar 21;17:46. doi: 10.1186/s12874-017-0316-6 (PMC5359986; doi:10.1186/s12874-017-0316-6)
Supplement: Additional file 1: — Study-Specific Guidelines: ICF-CY Linking Rules and Challenges. This file contains a summary of the ICF Linking Rules annotated with study-specific examples and a summary of solutions to commonly occurring challenges from Part I – Linking visual ability assessments to the ICF-CY. (DOCX 20 kb) [file 12874_2017_316_MOESM1_ESM.docx]

**Additional File 1: Study-Specific Guidelines: ICF-CY Linking Rules & Challenges**

Study Specific Guidelines based on:

- Cieza A, Geyh S, Chatterji S, Kostanjsek N, Ustun B, Stucki G: **ICF linking rules: an update based on lessons learned.** *J Rehabil Med* 2005, **37:**212-218.
- Cieza A, Fayed N, Bickenbach J, Prodinger B: **Refinements of the ICF Linking Rules to strengthen their potential for establishing comparability of health information.** *Disabil Rehabil* 2016**:**1-10.
- Fayed N, Cieza A, Bickenbach J: **Illustrating child-specific linking issues using the Child Health Questionnaire.** *American Journal of Physical Medicine & Rehabilitation* 2012, **91:**S189-S198.
- World Health Organization ICF Research Branch: **ICF eLearning Tool.** 2015.

| **Study Specific ICF-CY Linking Rules** | |
| --- | --- |
| Identification of linking units | |
| *i.* | Determine the type of information to be linked: *patient-oriented measure* (self-report, caregiver report, or health professional reported) or *clinical assessment*. |
| *ii.* | Identify linking unit(s). The linking unit of a measure answers the question: What is the item about?  The names of measures, the instructions, and subscale titles provide useful information to define the linking units.  *e.g. Item 17 from the CVI Questionnaire asks whether the child “Sits right in front of the television”. This item needs to be considered in the context of being an item in a measure screening for cerebral visual impairment. The item falls in the section of ‘Visual attitude’ and the subscale of ‘visual attention’. This item is not about ‘sitting’.*  For Patient-oriented measures:   - Refer to the item as it appears in the questionnaire - Identify response options of items that contain linking unit(s)   For Clinical assessments:   - Refer to the aim of the clinical assessment - Consider that the linking unit may change depending on the context in which the clinical assessment is used. |
| *iii.* | Identify any relationship between concepts: when there are more than two linking units the relationship between the units is also provided**.**  *e.g. Item 21 in the Functional Visual Questionnaire asks whether the child “Looks at a toy or object while reaching/moving hand towards it”. This item is about looking ‘whilst’ reaching. The relationship should be recorded.* |
| Linking of linking units to the ICF-CY | |
| *a.* | Select the appropriate code(s) to describe the linking unit:  Is the linking unit an element of Body Functions, Body Structures, Activities and Participation, or Environmental factors?  Which chapter within the selected domain is the most appropriate?  Which category within the selected chapter is the most precise? |
| *b.* | If the content of an item is not explicitly named in the corresponding ICF-CY category, then the “other specified” is linked. This code allows for coding of functioning that is not included within any of the other specific categories. When an “other specified” code is used, the specification has to be annotated. |
| *c.* | If the content of an item is insufficient to permit assignment of a more specific category, the “unspecified” is linked. The code has the same meaning as the second- or third-level term immediately above (b), without any additional information.  i.e. Use d199 Learning and applying knowledge, unspecified rather than d1 Learning and applying knowledge |
| *d.* | If the linking unit is an element of ‘Health condition’ the code HC is used. |
| *e.* | If the linking unit is an element of ‘Personal factors’ it would be considered to have a positive or negative influence on disability and functioning. To determine if a linking unit is a Personal factor ask: Can the linking unit be impaired, restricted or limited? If no, it is a personal factor. |
| *f.* | If the content of an item is unclear or too general to permit assignment of any category or component, the “nondefinable” (nd) is used. The perspective is documented as General Health (nd-gh), Quality of life (nd-qol), Physical health (nd-ph), Mental health (nd-mh), or Life satisfaction (nd-s). |
| *g.* | If the linking unit is not a Health condition, Body function/body structure, Activity, Participation, Environmental factor or Personal factor, it is “Not covered” (nc). |

***Summary of solutions to commonly occurring challenges from Part I: Linking assessments to the ICF-CY***

| **Challenge** | **Example item** | **Discussion and Solution** |
| --- | --- | --- |
| **Misleading terminology and unclear perspectives in items** | Item 1 in the Short CVI Questionnaire: Are there problems in seeing an object on patterned background? | As per linking guidelines no. *ii*, linkers need first to consider whether a linking unit is an element of Body Functions, Body Structures, Activities and Participation or Environmental factors, and then select the most appropriate chapter and code within that domain.  In this example, consideration needs to be given to the other available information i.e. this item is from a questionnaire about the presence of problems rather than an assessment of capacity or impairment, and there is mention of a potential environmental barrier. This item should be linked to the Activities and Participation domain.  Note: There is a mismatch between terminology defined by the ICF-CY and how it is used in clinical and research practice. i.e. “seeing” is defined by the ICF-CY in the Body Function domain at Chapter 2 ‘Sensory functions and pain’ with b210-b229 *Seeing and related functions*, however seeing is a term commonly used to described the purposeful/intentional use of vision, and d110 *Watching* is the Activity and Participation level code for ‘seeing’. |
| **Linking items about using vision to *d110 Watching* versus *d160 Focusing attention*** | Item 4 of the Callier Azusa Scale: May look at caregivers face when held. | The Activity and Participation domain codes of *d110 Watching* and *d160 Focusing attention* (including *d1600 Focusing attention on the human touch, face and voice*; *d1601 Focusing attention to changes in the environment*; and *d1608 Focusing attention, other specified*) are both highly relevant to measures of visual ability, however it is difficult to differentiate between these codes reliably.  In this example the item could be linked to *d110 Watching* and/or *d1600 Focusing attention on the human touch, face and voice,* as there is no exclusion criterion for linking to both codes. Items should be linked to the combined *d110 Watching* and/or *d160 Focusing attention* when they are about ‘using the sense of seeing intentionally to experience visual stimuli’ and ‘intentionally focusing on specific stimuli’. |
| **A code for ‘using vision’ could be added to almost every item included in this study** | Item 9a of the Visual Development subscale in the Callier Azusa Scale: May imitate movements of others. | To prevent excessive linking, and to maximise the usefulness of the results, items will only be linked when the linking unit is specifically about vision. All results will be considered with knowledge that the items are from measures of visual ability.  In the example provided on the left here, the item is about using vision to ‘imitate’, and this can be coded as *d130 Copying*. |
| **Interconnecting concepts need to be captured** | Item 21 in the Functional Visual Questionnaire: Looks at a toy or object while reaching/moving hand towards it. | As per the WHO ICF eLearning guidelines, when items include a relationship between concepts in a linking unit, the relationship should be recorded, e.g. “and”, “or”, “while”. If an item is about looking at something whilst reaching/moving hand towards it, the item is about two related concepts, and the connector e.g. “and” needs to be recorded. In this example the linking unit is about both looking and reaching together. Looking and reaching as separate but distinct constructs link to the Activity and Participation domain (e.g. *d110 Watching* + *d4452 Reaching*), whilst combining these constructs into one (e.g. eye-hand coordination) changes the construct to be about the ‘coordination’ and links to the Body Function domain (e.g. *b7602 Coordination of voluntary movements*). |
| **Linking items about the use of vision for discrimination within a task, action or everyday life** | Item 22 in the CVI Questionnaire: Does not recognize everyday objects such as an apple, bike, house, ball. | Items that are about the ability to visually recognize or perceive or discriminate, even within the situation of an everyday activity or task, are about the psychological function and will be linked to *b1561 Visual perception*.  Note: *b156 Perceptual functions* and *b210 Seeing functions* are exclusive categories. |
| **Linking items containing ‘visual situations’** | Item 3 in the Short CVI Questionnaire: Are there problems finding parents or friends in a crowd? | Assessments of visual ability contain items with reference to many ‘visual stimuli’ and ‘visual environments’. Together these could be termed ‘visual situations’ and they include:  type of visual stimuli, such as objects, people, pictures  characteristics of visual stimuli, such as size, colour, brightness, location, distance, moving or kept still  characteristics of physical environment, such as light, darkness, presence of other stimuli/clutter/distractions  assistance, such as glasses, sound clues  activities that provide a situation where vision may be used, such as reading  In order to determine whether and/or where a visual situation should be linked, linkers are encouraged to ask: I*s the visual situation a linking unit (being assessed)? Or is the visual situation described, in order for assessment of some other function or action (e.g. looking)?*  These ‘visual situations’ provide the characteristics or specifications of different levels of visual ability that provide key information in Part II of this study. In Part I, visual situation data which is not specifically linked should be recorded in the data extraction sheet under ‘additional information’. |
