# Supplementary material for: Methods for conceptualising ‘visual ability’ as a measurable construct in children with cerebral palsy
Source: BMC Med Res Methodol. 2017 Mar 21;17:46. doi: 10.1186/s12874-017-0316-6 (PMC5359986; doi:10.1186/s12874-017-0316-6)
Supplement: Additional file 2: — Results table for Body Function and Environmental factor codes. This file contains the tabulated results for assessment items linked to Body Function and Environmental factor codes. These results are not pertinent to Phase II in this study. (DOCX 21 kb) [file 12874_2017_316_MOESM2_ESM.docx]

**Additional File 2: Results tables for Body Function and Environmental factor codes**

| **Table A Body Function ICF-CY Categories identified in assessment tools** | | | | | | | | | | | | | | | | | | | | |
| --- | --- | --- | --- | --- | --- | --- | --- | --- | --- | --- | --- | --- | --- | --- | --- | --- | --- | --- | --- | --- |
|  | **Assessment tools** | | | | | | | | | | | | | | | | | | | |
| **ICF-CY Body Function Chpaters** | **ABCDEFV** | **Alimovic** | **CAS** | **CVI Q** | **CVI R** | **EDVA** | **FVQ** | **Hoyt** | **HSCS-PS** | **HUI-III** | **IDP** | **LVC** | **PreViAs** | **Sh CVI Q** | **SoGS** | **VAP-CAP** | **VSI** | **Wong** | **15-D** | **N** |
| b1 MENTAL FUNCTIONS | | | | | | | | | | | | | | | | | | | | |
| b114 Orientation functions |  |  |  |  |  |  |  |  |  |  |  |  | X |  |  |  |  |  |  | 1 |
| b122 Global psychosocial functions |  |  |  | X |  |  |  |  |  |  |  |  |  |  |  |  |  |  |  | 1 |
| b144 Memory functions |  |  |  | X | X |  |  |  |  |  |  |  |  |  | X |  |  |  |  | 3 |
| b147 Psychomotor functions |  |  |  | X |  |  |  |  |  |  |  |  |  |  |  |  | X |  |  | 2 |
| b156 Perceptual functions | X |  | X | X |  |  | X |  | X | X | X |  | X | X | X | X | X |  |  | 12 |
| b163 Basic cognitive functions | X |  |  |  |  |  |  |  |  |  |  |  | X |  |  | X |  |  |  | 3 |
| b167 Mental functions of language |  |  |  |  |  |  |  |  |  |  |  |  |  | X | X |  |  |  |  | 2 |
| b2 SENSORY FUNCTIONS & PAIN | | | | | | | | | | | | | | | | | | | | |
| b210 Seeing functions | X |  |  | X | X | X | X | X | X | X |  | X |  |  | X | X | X | X | X | 14 |
| b215 Functions of structures adjoining the eye | X |  | X |  |  |  |  |  |  |  |  |  | X |  | X |  |  |  |  | 4 |
| b230 Hearing functions |  |  |  |  |  |  |  |  |  |  |  |  | X |  |  | X |  |  |  | 2 |
| b7 NEUROMUSCULOSKELETAL AND MOVEMENT-RELATED FUNCTIONS | | | | | | | | | | | | | | | | | | | | |
| b750 Motor reflex functions | X |  | X |  | X | X |  |  |  |  | X | X |  |  |  |  | X |  |  | 7 |
| b760 Control of voluntary movement functions | X |  |  | X |  |  | X |  |  |  |  |  |  |  | X | X |  |  |  | 5 |

| **Table B Environmental factor ICF-CY Categories identified in assessment tools** | | | | | | | | | | | | | | | | | | | |  |
| --- | --- | --- | --- | --- | --- | --- | --- | --- | --- | --- | --- | --- | --- | --- | --- | --- | --- | --- | --- | --- |
|  | **Assessment tools** | | | | | | | | | | | | | | | | | | |  |
| **ICF-CY Environmental factor Chapters and Two-level classification** | **ABCDEFV** | **Alimovic** | **CAS** | **CVI Q** | **CVI R** | **EDVA** | **FVQ** | **Hoyt** | **HSCS-PS** | **HUI-III** | **IDP** | **LVC** | **PreViAs** | **ShCVI Q** | **SoGS** | **VAP-CAP** | **VSI** | **Wong** | **15-D** | **N** |
| **e1 PRODUCTS AND TECHNOLOGY** | | | | | | | | | | | | | | | | | | | |  |
| e125 Products and technology for communication |  |  |  |  |  |  |  |  | X | X |  |  |  |  |  |  | X |  | X | 4 |
| **e2 NATURAL ENVIRONMENT AND HUMAN-MADE CHANGES TO ENVIRONMENT** | | | | | | | | | | | | | | | | | | | | |
| e240 Light |  |  |  |  |  |  |  |  |  |  |  |  |  |  |  |  | X |  |  | 1 |
| **e3 SUPPORT AND RELATIONSHIPS** | | | | | | | | | | | | | | | | | | | | |
| e399 Support and relationships, unspecified |  |  |  |  |  |  |  |  |  |  |  |  |  |  |  |  |  |  | X | 1 |
